# Quality indicators of nutritional care practice in elderly care

**DOI:** 10.1007/s12603-017-0970-8

**Published:** 2017-09-23

**Authors:** Malin Skinnars Josefsson, M. Nydahl, I. Persson, Y. Mattsson Sydner

**Affiliations:** 1 Department of Food, Nutrition and Dietetics, Uppsala University, Box 560, 751 22 Uppsala, Sweden; 2 Department of Statistics, Uppsala University, Uppsala, Sweden

**Keywords:** Nutritional care practice, meal satisfaction, nutritional status, older adults, residential care homes

## Abstract

**Objectives:**

The aim is to explore the effects of antecedent, structural and process quality indicators of nutritional care practice on meal satisfaction and screened nutritional status among older adults in residential care homes.

**Design:**

Data for this Swedish cross-sectional study regarding older adults living in residential care homes were collected by i) a national questionnaire, ii) records from the quality registry Senior Alert, iii) data from an Open Comparison survey of elderly care in 2013/2014. The data represented 1154 individuals in 117 of 290 Swedish municipalities.

**Measurements:**

Meal satisfaction (%) and adequate nutritional status, screened by the Mini Nutritional Assessment Short Form (MNA-SF), were the two outcome variables assessed through their association with population density of municipalities and residents’ age, together with 12 quality indicators pertaining to structure and process domains in the Donabedian model of care.

**Results:**

Meal satisfaction was associated with rural and urban municipalities, with the structure quality indicators: local food policies, private meal providers, on-site cooking, availability of clinical/community dietitians, food service dietitians, and with the process quality indicators: meal choice, satisfaction surveys, and ‘meal councils’. Adequate nutritional status was positively associated with availability of clinical/community dietitians, and energy and nutrient calculated menus, and negatively associated with chilled food production systems.

**Conclusion:**

Municipality characteristics and structure quality indicators had the strongest associations with meal satisfaction, and quality indicators with local characteristics emerge as important for meal satisfaction. Nutritional competence appears vital for residents to be well-nourished.

## Introduction

Quality indicators are commonly used to improve nutritional care practice in elderly care (1-3), and a range of different strategies has been described (4-6). These strategies comprise a complex range from organisational to individual aspects, requiring a package of multilevel and multicomponent approaches (7). Previous studies have found quality indicators, such as the presence of adapted guidelines and policies, and the systematic education of staff, to be meaningful for the improvement of nutritional care practices (8-10). These quality indicators, together with nutritional competence and attitude, are described as vital for achieving interventions addressing malnutrition (11-13). Studies of links between food service practice and residents’ risk of malnutrition have further reported that overall meal satisfaction and menu cycle length, among other quality indicators, are associated with nutritional status (14). Examples of additional quality indicators addressed in interventions seeking to improve the food intake of older adults living in residential care homes are the sensory properties of food, mealtime logistics, nutrient density, variety and personalised meals (15, 16). Meal choice, as an example of a personalised meal, is reported to help increase body weight (17) and meal satisfaction (18). Findings from these and other studies describe a notable relationship between quality indicators of nutritional care practice such as food service satisfaction, nutritional status and food choice (7, 17-19).

Swedish elderly care, which includes nutritional care practice, is recognised for its comprehensiveness and high quality (20). Yet malnutrition, with its consequences for the individual and society, is also a reality in Sweden (21, 22). To address these problems, national authorities have provided guidelines that aim to improve the nutritional care practices for older adults (23, 24). Further, to improve quality and for the systematic prevention of malnutrition among older adults, and as a support for research, a national quality registry was launched in 2010 (25). Follow-ups on quality indicators of the performed and perceived overall quality of elderly care are conducted annually (26). Thus, guidelines and quality indicators for nutritional care are available, but there is a lack of knowledge concerning the outcomes of these supportive strategies. Elderly care, and how it is delivered, is the responsibility of local political councils, i.e. municipalities. There is extensive local autonomy and varying conditions resulting in diverse outcomes. National guidelines and quality indicators are interpreted at local level, where the responsibility for nutritional care is held.

In this paper, we address nutritional care practice with a focus on quality indicators related to food service, ranging from organisational set-ups to the older adults’ evaluation of meals. By doing so, this study seeks to present a holistic perspective, guided by Donabedians’ model of quality of care (27). In this model, quality indicators can be categorised into structure, process and outcome, creating a causal relationship between them. In the model, structure refers to quality indicators belonging to organisational, material and human resources. Process refers to what is actually done in giving and receiving care. Outcome quality indicators refer to the results of the care provided (27, 28). The model has been used in numerous studies over the years, some concerning nutritional care practice in hospital or residential care home settings at an institutional level (3, 29, 30). As a development of the model, it has been suggested that antecedents of care are also incorporated into the original framework as these factors are expected to affect the structure, process and outcome (31). The antecedents of care involve the personal characteristics and environmental context of an individual outside the care chain, e.g. socio-demographics and age. To our knowledge, there are no previous studies that have incorporated the whole chain of organisational conditions for nutritional care practice in elderly care. Hence, the aim is to explore the effect of antecedent, structural and process quality indicators of nutritional care practice in relation to the outcomes meal satisfaction and screened nutritional status among older adults in residential care homes.

## Methods

Framed by the Donabedian model, this paper draws on merged data including results from i) a national questionnaire, ii) records from the quality registry Senior Alert (25), and iii) data from an Open Comparison survey of elderly care (26).

**Figure 1:**
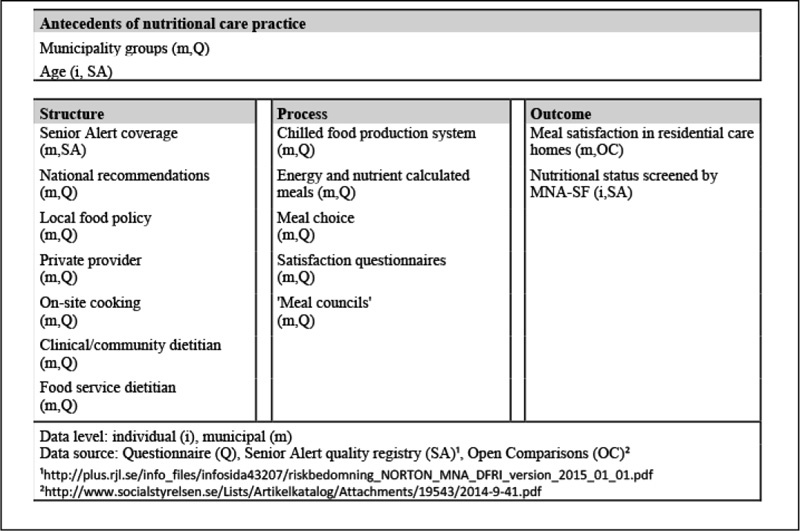
Source and data level of antecedents, structure, process and outcome quality indicators of nutritional care practice

Between November 2013 and January 2014, a comprehensive national questionnaire developed by the authors was sent to all Swedish municipalities (n=290). The questionnaire, which had been pilot-tested for comprehensibility by food service dietitians, was distributed by e-mail with a cover letter. The cover letter informed that responses were voluntary and, although not anonymous, would be confidential and that individual municipalities would not be identified in the presentation of the results. Two reminders were sent to non-repliers and additional telephone call reminders were made by the first author to encourage further responses, reaching a final response rate of 56% (n=162). Eleven questions were selected to serve as quality indicators in this paper, and questions with answers on an ordinal scale were dichotomised for the analysis (Figure 1). The questions placed as quality indicators in the structure domain of the Donabedian model were: if municipalities consult the national recommendations concerning meals for older adults provided by the National Food Agency (1= yes, 0= no), presence of a local food policy (1= yes, 0= no), if the meal provision is contracted out to a private provider (1= yes for all, for most, for half of the units, 0= no, for a few units); if the meals were cooked on-site at the residential care homes (1= yes in all, in most, in half of the units, 0= no, in a few units), and if community/clinical dietitian(s) (1= yes one, yes several, 0= no) and/or food service dietitian(s) (1=yes one, yes several, 0= no) were available. The following questions were placed in the process domain: whether a cook-chill food production system was used (1= yes for all, most, half of the units, 0= no, for a few units), if meals were energy and nutrient calculated (1= yes all meals, lunch and dinner 0= lunch or dinner, no), if meal choices were offered for one or two main meals (1= yes for all, most, half of the units, 0= no, for a few units), if residents were frequently asked about their satisfaction with meals through questionnaires (1= yes, 0= no), and if meetings with representatives from the residents were held regularly where the menu and quality of food were discussed, called ‘meal councils’ in this study (1= yes, 0= no).

Data from the national quality registry Senior Alert (25) were anonymised on an individual but not on a municipal level, in order to make the data connectable to other sources in the analysis. If there were multiple registrations of an individual in the Senior Alert registry, only the first was included along with those registrations pertaining to municipalities participating in the questionnaire. The requested data covered the period January to March 2014 and comprised the areas of nutritional risk assessment of older adults residing in residential care homes. The measures in the quality registry are based on evidence. In 2013, all municipalities with the exception of four (n=286) contributed to the national quality registry although with varying coverage, i.e. varying proportions of their residents being included (32). The validated risk assessment tool used in the registry is the Mini Nutritional Assessment Short Form (MNA-SF) (33). According to MNA-SF screening, a person is considered malnourished with scores 0-7, at risk of malnutrition with scores 8-11 and well-nourished with scores 12-14. From the Senior Alert registry, the MNA-SF scores were placed in the outcome domain (1=well-nourished, 0= malnourished or at risk), and coverage in the registry was placed as a structure indicator (continuous variable) (Figure 1). To enable calculation of the MNA-SF score, only registrations with complete values for all items on the MNA-SF were included in the analysis.

Data from the Open Comparisons survey of elderly care in 2014 was obtained from the National Board of Health and Welfare (NBHW) (26). These self-reported data can be freely accessed at municipal level. All municipalities (n=290) participated in the 2014 survey, however some values were missing due to partially low internal response rates. For this paper, data on residents’ satisfaction with meals (%), represented by the question ‘In general, how does the food taste?’, was selected from the survey and placed as a second outcome indicator (Figure 1).

In order to control for antecedents of nutritional care practice, residents’ age and the population density of municipalities were the first indicators to be put into the model. Municipalities were grouped into rural, urban, and city using a classification based on population density, size and proximity to population agglomerations (34). Dummies were created for rural and urban municipality groups. Information on residents’ age was collected from the quality registry Senior Alert.

**Table 1:**
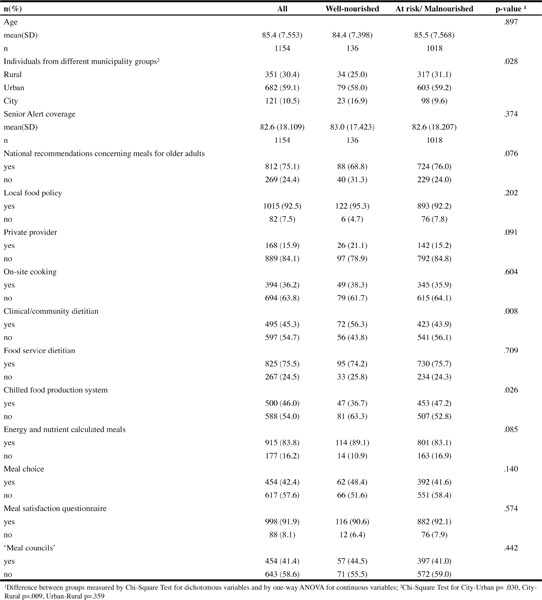
Distribution of antecedent, structure and process quality indicators of nutritional care practice based on nutritional status (wellnourished or at risk/malnourished), (n=1154)

**Table 2:**
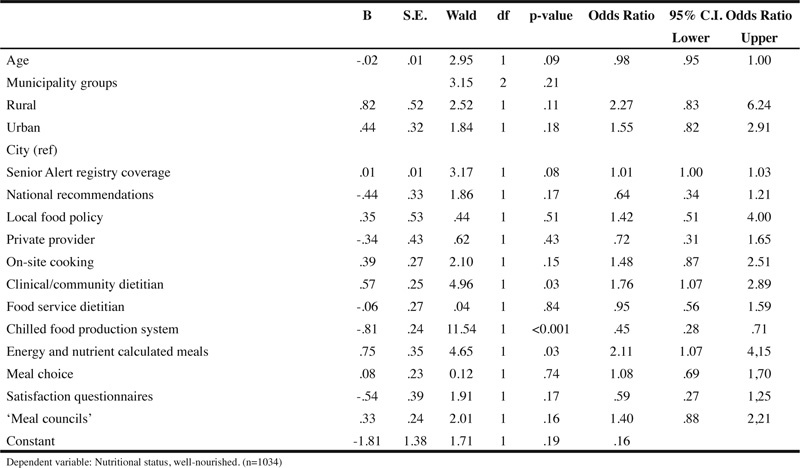
Antecedents and quality indicators of nutritional care practice’s association with being well-nourished

### Data analysis

Data were analysed using the statistics program IBM SPSS version 22.0. After aggregating data from the different sources and excluding cases with missing values crucial for the analysis, the dataset contained 1154 individuals representing 117 of 290 Swedish municipalities. Descriptive statistics of the sample were compared by nutritional status (well-nourished and malnourished/at risk). Comparison of individual indicators on nutritional status was performed using Pearson’s χ2-test for categorical variables and one-way ANOVA for continuous variables. Before conducting a hierarchal regression analysis, Pearson’s correlation analysis was performed to investigate the bivariate relationship between the dependent variable satisfaction with meals and the explanatory variables. The hierarchical regression analysis created models introducing explanatory variables in the following steps: 1) antecedents of nutritional care practice, 2) structure quality indicators, and 3) process quality indicators. A binomial logistic regression was performed to ascertain the effects of structure and process quality indicators on the likelihood of older adults in residential care homes being screened as having adequate nutritional status (being well-nourished). In the model, the dependent variable was screened nutritional status (well-nourished or not). The statistical significance level for all analyses was set at 0.05 (significant result if p < .05). Multiple tests have been performed, which means that the total significance level is greater than the 5% used in a single test; the significance of the different test results must therefore be interpreted with care.

## Results

Table 1 shows the distribution of the antecedents of nutritional care practice, structure and process quality indicators for the nutritional status groups screened by MNA-SF. Rural, urban and city municipalities differed significantly regarding the proportion of individuals classified as well-nourished or at risk/malnourished (p=.028), with city municipalities having a higher proportion of well-nourished older adults. Availability of a clinical/community dietitian (p=.008) and chilled food production systems (p=.026) showed significant differences in their association with being well-nourished. Table 2 shows that one structure and two process indicators had a statistically significant association with screened nutritional status (wellnourished). The availability of a clinical/community dietitian was positively associated, odds ratio 1.76, and offering energy and nutrient calculated meals more than doubled the odds of being well-nourished, odds ratio 2.11, while the use of a chilled food production system was negatively associated with being well-nourished, odds ratio 0.45. The full model containing all indicators was statistically significant (χ2(15) = 38.441, p = .001), and explained approximately 7% (Nagelkerke R^2^) of the variance in scoring of being well-nourished by MNA-SF.

Table 3 describes the bivariate correlations between meal satisfaction and the explanatory variables. The strongest correlation was between meal satisfaction and food service dietitian (r=.273, p<.01), but overall the correlations with meal satisfaction were weak (r<±.3) although a majority had significant correlations. Based on this, we investigated the simultaneous relationship between a combination of explanatory variables and meal satisfaction. Overall, the correlations between the explanatory variables were also weak, which indicates a lack of multicollinearity problems. The moderate correlation between private provider and clinical/community dietitian (r=.440, p<.01) was the strongest correlation found. Table 4 summarises the results of the hierarchical regression analysis for meal satisfaction. In model 1, municipality groups and age together explained 3.1% of the total variance of satisfaction with meals for the study sample, of which residents in residential care homes in rural and urban municipalities were significantly more likely to be satisfied than residents in city municipalities. Model 2 added structure quality indicators and explained an additional 11.4% of the variance. Older adults in municipalities where private providers supplied the meals, meals were cooked at site and a food service dietitian was available, were more likely to be satisfied with meals, while availability of a clinical/community dietitian had a negative association with meal satisfaction. As a final step, process quality indicators were entered, producing a model that added another 3.7% of the variance of meal satisfaction being explained. This third and final model showed that older adults living in residential care homes using a chilled food production system, offering energy and nutrient calculated meals, and meal choices, were significantly less likely to be satisfied with meals. Local food policies entered in model 2, became significantly associated with meal satisfaction in the final model. The total variance explained by the full model was 18.2% (F (14,1000) = 31.085, p < .0001).

## Discussion

Municipality characteristics (rural, urban or city) and the structure and process quality indicators in the Donabedian model had a more pronounced association with the outcome of meal satisfaction than screened nutritional status among older adults living in residential care homes. Meal satisfaction was positively associated with quality indicators pertaining to structure. These were, a local food policy, private provider, on-site cooking, and availability of food service dietitians. Meal satisfaction was negatively associated with availability of clinical/community dietitians, and all but one process indicators. For the outcome variable screened adequate nutritional status, two quality indicators were positively significant: availability of a clinical/community dietitian, and offering energy and nutrient calculated meals, while chilled food production systems was negatively associated.

**Table 3:**
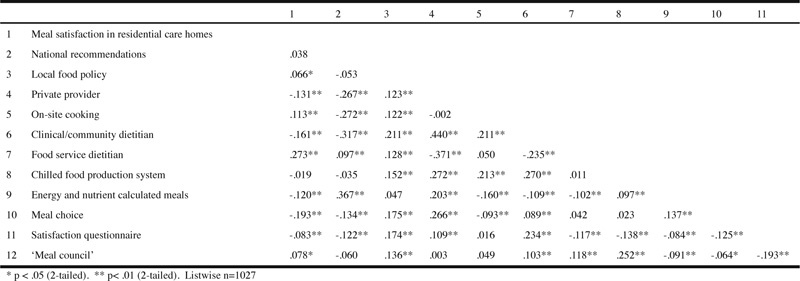
Pearson’s product-moment correlations between bivariate quality indicators and meal satisfaction in residential care homes

**Table 4:**
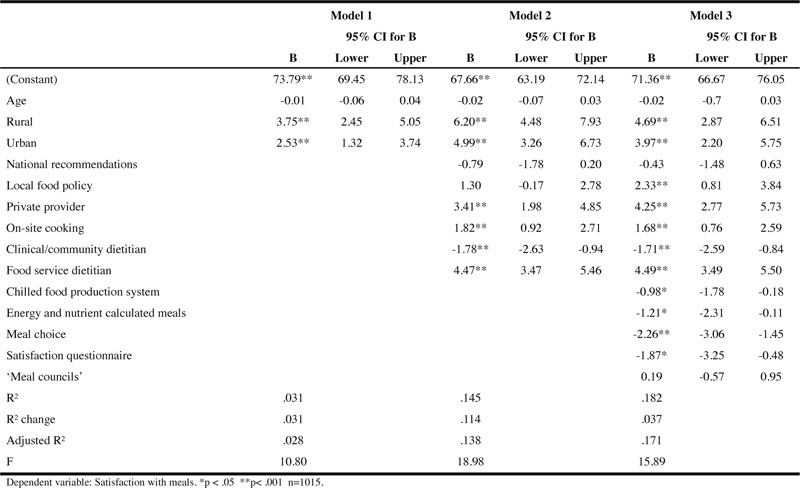
Hierarchical regression models on meal satisfaction in residential care homes

In our study of quality indicators of nutritional care practice, local food policies and availability of clinical/community and food service dietitians stood out as considerable contributors. These structure quality indicators commonly provide the basis for the application of process quality indicators, such as choice of meals or energy and nutrition calculated menus. Overall, structure quality indicators were positively associated with meal satisfaction while those related to process were negatively associated. These results contradict the findings of Kajonius and Kazemi (35), who, in their study based on similar data, found that overall satisfaction with elderly care was determined by factors pertaining to process. However, as they also discuss, there is a need to evaluate how the structure and process quality indicators have been operationalised and chosen, and which outcome variables have been studied. In this study, our aim was to present a holistic perspective ranging from organisational set-ups to individual evaluation of meals. However, the quality indicators chosen focus on aspects of food service, representing one of many areas in this multidimensional and complex field of nutrition care practice. This makes it difficult not only to compare different studies but also to evaluate the impact of the results between structure and process domains. Instead, we suggest focusing on their joint effect as a link to the outcome variable when interpreting the results, which we regard as contributing to this broad field.

Local food policies that are adapted to local conditions and produced within the organisation related positively to meal satisfaction, although they did not significantly associate with adequate nutritional status. These results are in line with a study by Meijer et al, where no structural quality indicators were found to be good predictors of malnutrition over time (29). However, with the objective of improving daily care and food service satisfaction of older adults, a need for practical guidelines has been underscored in studies by e.g. Volkert and Wright et al. (36, 37), along with a need for ensuring their effectiveness (38).

One of the positive associations with meal satisfactionv was cooking on-site. Through providing greater flexibility, cooking on-site is beneficial for the individual resident making personal requests easier to address than if meals come ready prepared (39). One plausible explanation for the negative association between chilled food production systems and both outcome variables is that a chilled food production system often requires reduced transportation frequency due to long distances, thus limiting flexibility. Another explanation could be a perceived impaired sensory quality of the food since it is chilled and reheated (40). Further, the negative association between meal satisfaction and energy and nutrient calculation of meals is puzzling but, like the findings of Wright et al, might be explained by a desire for comfort foods on the elderly care menu (36); comfort food that does not necessarily meet nutritional requirements. In addition, the classic dishes served might not contain the ‘traditional’ ingredients and condiments due to lack of awareness or to making restrictions in order to meet nutritional requirements. Hence, the traditional culinary rules are not followed and the symbolic meaning of the dish is lost (41).

The quality indicator meal choice in our study also associated negatively with meal satisfaction, and contradicts the results of Wright et al (36). As described by Kenkmann and Hooper, choices could be withheld from residents during hectic times, or that residents not always made choices even if possible, but instead trusted care staff to make the choices for them (42). With the strongest association, the importance of food service dietitians for the likelihood of older adults being satisfied with meals, is also confirmed in this study, as is the importance of clinical/community dietitians for adequate nutritional status, while an explanation for the negative association between clinical/community dietitians and meal satisfaction is inexplicable.

Although challenging to assess, satisfaction as a quality indicator is emphasised by Donabedian as a core value in care (28). Meal satisfaction as a quality indicator is presented in numerous studies as influencing dietary intake and overall satisfaction (36, 43, 44). However, the problem of finding appropriate measurement strategies has been discussed, since older adults living in residential care homes might have difficulty expressing themselves, due to suffering from dementia or other cognitive impairments (45). These residents are also less inclined to complain about meals (19).

Even if the MNA-SF is a screening tool and therefore does not guarantee qualitative nutritional care, it is valuable for early recognition of risks among older adults where there is an intention to generate dietetic interventions (46). Since it is utilised by a variety of healthcare professionals, there may be differences in interpretation of the screening tool with the risk of obtaining misleading results. However, MNA-SF is considered to be a robust screening tool regardless of the training or professional background of those using it (47). There is no doubt that nutritional status as an outcome indicator is fundamental and essential to consider due to the considerable prevalence of malnutrition in elderly care (37, 48-50).

The results in this study rely on self-reported data, which can be considered a limitation and a reliability risk. The annual open comparison surveys are directed to the older adult, but instructions encourage responders to ask for assistance if they need help in responding. According to the NBHW, those reporting poor health in the survey were more inclined to ask for help and these responses were more negative (26). We do not know the proportion of independent responders in our study. Strengths of this study include the overall comprehensiveness through the use of rich nationwide databases with evidence-based settings and the practical implications for elderly care organisations. Further, a potential drawback of our data is that it ranged from an individual to a municipal level. This was however a prerequisite for performing the various analyses of the conceptual model including the whole chain from antecedents of nutritional care practice to outcomes. Future research would benefit from incorporating additional quality indicators in order to further understand important associations and predictors of nutritional care practice, and the outcome of interventions building on this knowledge.

## Conclusion

This study contributes insight into the association of quality indicators of nutritional care practice and their link to meal satisfaction and screened nutritional status, focusing on aspects of food service in elderly care. Considering the nature of the Donabedian model, which implies a causal chain with process indicators building on structure, the positive association between structure quality indicators and the negative association with process quality indicators regarding meal satisfaction can be interpreted in several ways. One is that the older adults are confident in the local organisation to provide optimal nutritional care, and that active participation such as choosing meals, or part taking in questionnaires, is unwanted. The other interpretation is that the negative association with process indicators is a sign of system malfunction, where for example meal choices might be withheld from the residents and hence a service only in theory. Further, for meal satisfaction the significant associations with quality indicators were many, whereas significant associations with screened nutritional status were few. This discrepancy might be explained by the different data levels for the two outcome variables. Compiling associations with both of them, clinical/community dietitians as well as food service dietitians stand out as central, and the chilled food production system appear to be an unhelpful factor in the nutritional care practice with its negative association with both meal satisfaction and being well-nourished.

*Acknowledgements*: We thank the survey participants and the quality registry Senior Alert for the data and ‘Stiftelsen Kronprinsessan Margarets minne’ for funding the study.

*Conflict of interests*: There was no conflict of interest for any of the authors.

*Ethical approval*: Ethical approval was received by an advisory statement from the Regional Ethical Review Board of Medical Sciences in Uppsala (ref. no. 106 2013/386/1).

*Authorship*: MSJ was responsible for the development of the study, data collection, analysis, and drafting of the manuscript. YMS and MN supervised the study and contributed to drafting and editing the manuscript. IP was involved in the data analysis and editing the manuscript. All authors approved the final version of the paper.

## References

[1] van der Pols-Vijlbrief R, Wijnhoven HAH, Schaap LA, Terwee CB, Visser M (2014). Determinants of protein-energy malnutrition in community-dwelling older adults: A systematic review of observational studies. Ageing Res Rev.

[2] Dupuy C, de Souto Barreto P, Ghisolfi A, Guyonnet S, Dorigny B, Vellas B (2016). Indicators of oral nutritional supplements prescription in nursing home residents: A cross-sectional study. Clin Nutr.

[3] van Damme N, Buijck B, van Hecke A, Verhaeghe S, Goossens E, Beeckman D (2015). Development of a quality of meals and meal service set of indicators for residential facilities for elderly. J Nutr Health Aging.

[4] Abbott RA, Whear R, Thompson-Coon J, Ukoumunne OC, Rogers M, Bethel A (2013). Effectiveness of mealtime interventions on nutritional outcomes for the elderly living in residential care: A systematic review and meta-analysis. Ageing Res Rev.

[5] Tamura BK, Bell CL, Masaki KH, Amella EJ (2013). Factors Associated with weight loss, low BMI, and malnutrition among nursing home patients: A Systematic Review of the Literature. J Am Med Dir Assoc.

[6] Kimber K, Gibbs M, Weekes CE, Baldwin C (2015). Supportive interventions for enhancing dietary intake in malnourished or nutritionally at-risk adults: a systematic review of nonrandomised studies. J Hum Nutr Diet.

[7] Vucea V, Keller HH, Ducak K (2014). Interventions for improving mealtime experiences in long-term care. J Nutr Gerontol Geriatr.

[8] Westergren A, Hedin G (2010). Do study circles and a nutritional care policy improve nutritional care in a short-and long-term perspective in special accommodations. Food Nutr Res.

[9] van Nie NC, Meijers JMM, Schols JMGA, Lohrmann C, Spreeuwenberg M, Halfens RJG (2014). Do structural quality indicators of nutritional care influence malnutrition prevalence in Dutch, German, and Austrian nursing homes. Nutrition.

[10] Pezzana A, Cereda E, Avagnina P, Malfi G, Paiola E, Frighi Z (2015). Nutritional care needs in elderly residents of long-term care institutions: Potential implications for policies. J Nutr Health Aging.

[11] Leslie WS (2011). Improving the dietary intake of frail older people. Proc Nutr Soc.

[12] Lorefält B, Andersson A, Wirehn AB, Wilhelmsson S (2011). Nutritional status and health care costs for the elderly living in municipal residential homes-An intervention study. J Nutr Health Aging.

[13] Beck A, Andersen UT, Leedo E, Jensen LL, Martins K, Quvang M (2015). Does adding a dietician to the liaison team after discharge of geriatric patients improve nutritional outcome: a randomised controlled trial. Clin Rehabil.

[14] Carrier N, Ouellet D, West GE (2007). Nursing home food services linked with risk of malnutrition. Can J Diet Pract Res.

[15] Keller H, Beck AM, Namasivayam A (2015). Improving food and fluid intake for older adults living in long-term care: A research agenda. J Am Med Dir Assoc.

[16] Hedman S, Nydahl M, Faxén-Irving G (2016). Individually prescribed diet is fundamental to optimize nutritional treatment in geriatric patients. Clin Nutr.

[17] Crogan NL, Dupler AE, Short R, Heaton G (2013). Food choice can improve nursing home resident meal service satisfaction and nutritional status. J Gerontol Nurs.

[18] Kenkmann A, Price GM, Bolton J, Hooper L (2010). Health, wellbeing and nutritional status of older people living in UK care homes: an exploratory evaluation of changes in food and drink provision. BMC geriatrics..

[19] Crogan NL, Short R, Dupler AE, Heaton G (2015). The influence of cognitive status on elder food choice and meal service satisfaction. Am J Alzheimer’s Dis Other Dem.

[20] Olsen GM (2013). What’s ‘left’ in the ‘Garden of Sweden’. Int J Health Services.

[21] Söderström L, Thors Adolfsson E, Rosenblad A, Frid H, Saletti A, Bergkvist L (2013). Mealtime habits and meal provision are associated with malnutrition among elderly patients admitted to hospital. Clin Nutr.

[22] Borgström Bolmsjö B, Jakobsson U, Mölstad S, Östgren CJ, Midlöv P (2015). The nutritional situation in Swedish nursing homes -A longitudinal study. Archives Gerontol Geriatr.

[23] National Board of Health and Welfare. Näring för god vård och omsorg: en vägledning för att förebygga och behandla undernäring [Nutrition for good care: a guide to prevent and treat malnutrition]. Stockholm: Socialstyrelsen; 2011.

[24] Swedish National Food Agency. (2011). Bra mat i äldreomsorgen.

[25] Edvinsson J, Rahm M, Trinks A, Höglund PJ (2015). Senior alert: A quality registry to support a standardized, structured, and systematic preventive care process for older adults. Qual Manag Health Care.

[26] National Board of HealthWelfare. Öppna jämförelser 2014. (2014). Vård och omsorg om äldre: jämförelser mellan kommuner och län.

[27] Donabedian A (1966). Evaluating the quality of medical care. Milbank Mem Fund Qual.

[28] Donabedian A (1988). The quality of care: How can it be assessed. JAMA.

[29] Meijers JM, Tan F, Schols JM, Halfens RJ (2014). Nutritional care; do process and structure indicators influence malnutrition prevalence over time. Clin Nutr.

[30] Persenius M, Hall-Lord ML, Wilde-Larsson B, Carlsson E (2015). Clinical nursing leaders’ perceptions of nutrition quality indicators in Swedish stroke wards: A national survey. J Nurs Manag.

[31] Coyle YM, Battles JB (1999). Using antecedents of medical care to develop valid quality of care measures. Int J Qual Health Care.

[32] Henriks G, Edvinsson J, Trinks A (2013). Årsrapport 2013 Senior alert.

[33] Kaiser MJ, Bauer JM, Ramsch C, Uter W, Guigoz Y, Cederholm T (2009). Validation of the Mini Nutritional Assessment short-form (MNA®-SF): A practical tool for identification of nutritional status. J Nutr Health Aging.

[34] Growth Analysis. (2014). Better statistics for better regional and rural policy. Report.

[35] Kajonius PJ, Kazemi A (2016). Structure and process quality as predictors of satisfaction with elderly care. Health Soc Care Community.

[36] Wright ORL, Connelly LB, Capra S, Hendrikz J (2013). Determinants of foodservice satisfaction for patients in geriatrics/rehabilitation and residents in residential aged care. Health Expectations.

[37] Volkert D (2013). Malnutrition in older adults-urgent need for action: A plea for improving the nutritional situation of older adults. Gerontology.

[38] Woods JL, Walker KZ, Iuliano-Burns S, Strauss BJ (2009). Malnutrition on the menu: Nutritional status of institutionalised elderly Australians in low-level care. J Nutr Health Aging.

[39] Burger C, Kiesswetter E, Gietl A, Pfannes U, Arens-Azevedo U, Sieber CC (2016). Size matters! Differences in nutritional care between small, medium and large nursing homes in Germany. J Nutr Health Aging.

[40] Mavrommatis Y, Moynihan PJ, Gosney MA, Methven L (2011). Hospital catering systems and their impact on the sensorial profile of foods provided to older patients in the UK. Appetite.

[41] Sydner Mattsson Y, Fjellström C (2006). The meaning of symbols of culinary rules. J Foodservice.

[42] Kenkmann A, Hooper L (2012). The restaurant within the home: Experiences of a restaurantstyle dining provision in residential homes for older people. Qual Ageing Older Adults.

[43] Barnes S, Wasielewska A, Raiswell C, Drummond B (2013). Exploring the mealtime experience in residential care settings for older people: An observational study. Health Soc Care Community.

[44] Pizzola L, Martos Z, Pfisterer K, de Groot L, Keller H (2013). Construct validation and test–retest reliability of a mealtime satisfaction questionnaire for retirement home residents. J Nutr Gerontol Geriatr.

[45] Simmons SF, Cleeton P, Porchak T (2009). Resident complaints about the nursing home food service: Relationship to cognitive status. J Gerontol Ser B Psychol Sci Soc Sci.

[46] Kellett J, Kyle G, Itsiopoulos C, Naunton M (2016). Nutrition screening practices amongst Australian residential aged care facilities. J Nutr Health Aging.

[47] Win AZ, Ceresa C, Arnold K, Allison TA (2017). High prevalence of malnutrition among elderly veterans in home based primary care. J Nutr Health Aging.

[48] Törmä J, Winblad U, Cederholm T, Saletti A (2013). Does undernutrition still prevail among nursing home residents. Clin Nutr.

[49] Verbrugghe M, Beeckman D, Van Hecke A, Vanderwee K, Van Herck K, Clays E (2013). Malnutrition and associated factors in nursing home residents: A cross-sectional, multi-centre study. Clin Nutr.

[50] Johansson L, Wijk H, Christensson L (2017). Improving nutritional status of older persons with dementia using a national preventive care program. J Nutr Health Aging.

